# Advances on the early cellular events occurring upon exposure of human macrophages to aluminum oxyhydroxide adjuvant

**DOI:** 10.1038/s41598-023-30336-1

**Published:** 2023-02-23

**Authors:** J.-D. Masson, G. Badran, M. A. Domdom, R. K. Gherardi, B. Mograbi, F. J. Authier, G. Crépeaux

**Affiliations:** 1 Univ Paris Est Créteil, INSERM, IMRB, U955 GMC, 7 Avenue du General De Gaulle, 94010 Créteil, Maisons-Alfort, France; 2 Centre Antoine Lacassagne, CNRS, INSERM, IRCAN, FHU-OncoAge, Université Côte d’Azur, 06107 Nice, France; 3 Service d’Histologie/Centre Expert de Pathologie Neuromusculaire, AP-HP, Hôpitaux Universitaires Henri Mondor, 94010 Créteil, France; 4 Ecole Nationale Vétérinaire d’Alfort, IMRB, 94700 Maisons Alfort, France

**Keywords:** Autophagy, Pharmacodynamics, Inflammation, Adjuvants

## Abstract

Aluminum compounds are the most widely used adjuvants in veterinary and human vaccines. Despite almost a century of use and substantial advances made in recent decades about their fate and biological effects, the exact mechanism of their action has been continuously debated, from the initial “depot-theory” to the direct immune system stimulation, and remains elusive. Here we investigated the early in vitro response of primary human PBMCs obtained from healthy individuals to aluminum oxyhydroxide (the most commonly used adjuvant) and a whole vaccine, in terms of internalization, conventional and non-conventional autophagy pathways, inflammation, ROS production, and mitochondrial metabolism. During the first four hours of contact, aluminum oxyhydroxide particles, with or without adsorbed vaccine antigen, (1) were quickly recognized and internalized by immune cells; (2) increased and balanced two cellular clearance mechanisms, i.e. canonical autophagy and LC3-associated phagocytosis; (3) induced an inflammatory response with TNF-α production as an early event; (4) and altered mitochondrial metabolism as assessed by both decreased maximal oxygen consumption and reduced mitochondrial reserve, thus potentially limiting further adaptation to other energetic requests. Further studies should consider a multisystemic approach of the cellular adjuvant mechanism involving interconnections between clearance mechanism, inflammatory response and mitochondrial respiration.

## Introduction

The most frequently used adjuvants in commercial human and veterinary vaccines are aluminum (Al) salts. The word “adjuvant” comes from the latin word “*adjuvare*” meaning “to help”. Adjuvants enhance the immunogenicity of highly purified antigens that have insufficient immunostimulatory abilities^1^. Since Glenny and colleagues have demonstrated potassium Al sulfate (“alum”) adjuvanticity in 1926^2^, Al salts have been one of the main adjuvants used in licensed vaccines for approximately 70 years^1^. After Glenny’s discovery, alum was optimized selecting the best anions for preparation and changing antigens precipitation to adsorption leading to actual aluminum based adjuvants (ABAs), the Al oxyhydroxide (AH) at the end of the 1940s, the Al hydroxyphosphate later on^3^, and more recently the Al hydroxyphosphate sulfate.

Despite their longstanding use, the exact mechanisms of action induced by vaccine adjuvants are not fully understood, and this important question still remains to be elucidated^4,5^. Historically, the first proposed mechanism, described by Glenny himself, was the so-called “depot-theory” explaining the adjuvant effect of alum by antigen retention at the surface of slowly soluble adjuvant particles allowing prolonged antigen release from the injection site^6^. The slow antigen release could then activate recruitment and maturation of antigen-presenting cells (APCs)^7^ and enhance the expression of major histocompatibility complex class II molecules^8–10^. This mechanism has been challenged by demonstration that quick removal of the injection site containing the ABA depot did not affect the immune response^11,12^, suggesting other mechanisms at play.

In vitro*,* ABAs can directly stimulate components of the innate immune response such as those of the complement system^13^ and enhance the expression of chemotactic proteins^14–16^ that attract immune cells even without antigen^15^, leading to a new approach of ABAs action based on specific biological effects. The direct stimulation of innate immunity by ABAs induces a strong pro-inflammatory response at the injection site with activation of the NLRP3 inflammasome and production of interleukin (IL) IL-1β, IL-18 and IL-33^17–19^. It has been proposed that NLRP3 activation could result from destabilization of phagolysosomes by ABAs but this explanation is still in debate because of the lack of reproducibility^20^. Other possibilities include cellular Ca^2+^ and K^+^ efflux and reactive oxygen species (ROS) generation by mitochondria overflowing the scavenging ability of autophagy, the cellular homeostasis keeper^21–25^. However, the exact molecular signaling pathway of NLRP3 activation by ABAs is not well established and remains in debate. Indeed, absolute requirement of NLRP3 activation for the ABAs adjuvant effect has not been confirmed by all studies, a few of them reporting limited impact of either NLRP3 or caspase-1 deficiency on antibody production^19,26^.

More recently, the ABAs adjuvant effect was associated with their cytotoxicity possibly due to their crystalline structure^27^. This association was based on the immune stimulating properties of the crystallized uric acid acting as a danger associated molecular pattern which is highly pro-inflammatory after cell recognition^27,28^. After phagocytosis, the crystalline structure of ABAs (especially for AH^29^) could disrupt membranes leading to inflammation and cell death by both apoptosis and pyroptosis^30^, APCs maturation and therefore antibody production. This assumption was further supported by host DNA release at the AH injection site^31^. Host DNA is a potent stimulator which, like ABAs, induces a Th2-biased immune response promoting IgE production^14,32,33^. Here again, however, discrepant results have been reported. Some data suggested that the host DNA release following intramuscular ABAs injection may not be required for immune cell migration to the draining lymph node^31^. This point indicates that the effects of ABAs through DNA release are still not fully characterized and deserve further investigations.

Considering the remaining uncertainties about the fine biological effects of ABAs, we thought valuable to explore the early responses of primary Peripheral Blood Mononuclear Cells (PBMCs) obtained from healthy donors to AH, with and without adsorbed antigen, in terms of (1) cellular internalization of particles; (2) canonical autophagy (macroautophagy) as homeostasis keeper and LC3-Associated Phagocytosis (LAP) as a non-canonical autophagy pathway crucially contributing to immune responses through combination of the molecular machinery of phagocytosis with the autophagy machinery; (3) inflammatory response; iv) ROS production; and v) mitochondrial metabolism as a key compartment for energy production and possible source of ROS. PBMCs were chosen as key player of immune response. Indeed these cells are attracted into injured muscle by resident muscle macrophages and then become macrophages and monocytes-derived dendritic cells able to take up ABAs particles before migrating to the draining lymph nodes^10,34^.

Advances on the early AH adjuvant effects on human macrophages came from this systematic exploratory approach, mainly including implication of LAP balancing macroautophagy for cellular clearance, early release of tumor necrosis factor alpha (TNF-α), and negative impact on mitochondrial function.

## Results

### Identification of intracellular AH

**Figure 1:**
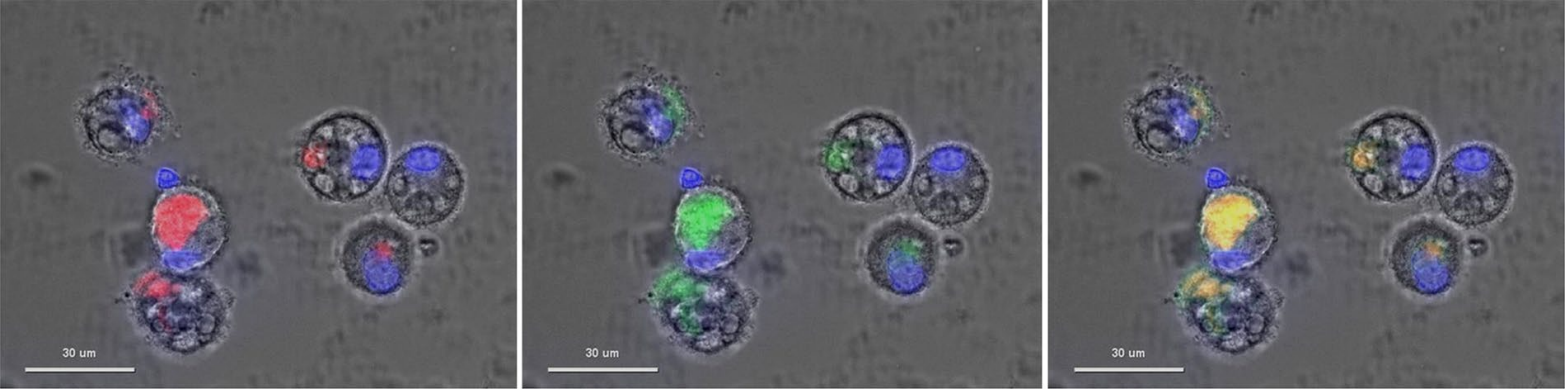
Example of fluorescence microscopy showing lumogallion signal in red (left panel), lysotracker signal in green (central panel), and merged signals (right panel) into differentiated PBMCs exposed 4 h with lumogallion-stained-AH. Scale bars : 30 µm.

To visualize AH internalization by PBMCs, cells were exposed to aluminum particles stained with lumogallion, and then fluorescent signal was detected 4 h after exposure. DAPI fluorescence allowed the identification of living cells by the presence of blue nuclei (used as background for the other fluorescent signals). Fluorescence microscopy of lumogallion-stained-AH and lysotracker showed that AH was internalized by differentiated PBMCs. Specific red fluorescence of lumogallion was clearly identified in the cytoplasm. Moreover, lumogallion fluorescence was observed very close to the green fluorescence of lysotracker (main Pearson R: 0.98; variation from 0.94 to 0.99 according to the individual; *p* < 0.01) leading to a yellow-orange signal on the merged signals pictures (Fig. 1 more detailed in Supplementary 1). This visual proximity between AH and lysosomes seems to indicate that Al particles were picked up by an internalization mechanism such as phagocytosis or LAP leading to an attempt to destroy the internalized compound by fusion with the lysosome.

### AH modulates autophagy and LAP

We then examined the response of two mechanisms, autophagy and LAP, in AH-exposed PBMCs, by assessing the expression of key proteins using Western blot analysis. As previously discussed in a specific review^35^, LC3-II protein level can be positively associated to the presence of autophagosomes; LC3-II/LC3-I is representative to autophagosomes de novo production; and SQSTM1/p62 protein level is inversely indicating the level of autophagolysosome degradation after fusion with the lysosome.

As a first step, autophagy modulators chloroquine (CQ) and rapamycin (Rapa), respectively inhibitor and activator, were used to test autophagy function of PBMCs unexposed to AH. After 4 h of treatment, CQ significantly increased the level of LC3-II, and also increased the LC3-II/LC3-I ratio which is in keeping with autophagosomes accumulation after negative regulation of autophagy by lack of degradation. Rapa produced a significant decrease of LC3-II and SQSTM1/p62 which are considered to indicate an active autophagy flux (Table 1).

**Table 1:** Autophagy/LAP related proteins quantification into differentiated PBMCs exposed 4 h to several treatments.

Treatment	Vehicle	CQ	Rapa	AH	AH + CQ	AH + Rapa	V	V + CQ	V + Rapa	Friedman	
										Χ^2^	*p*
LC3-II	1.00 (0.79–1.79)	2.69 (2.19–3.43) ▲	0.76 (0.40–1.19) ▲ △	2.24 (1.81–3.33) ▲ ■	4.14 (3.14–4.95) △ †	1.66 (1.28–2.28) ■ †	1.24 (1.03–1.52) △ ■ †	3.24 (2.52–3.53) ♦	1.55 (0.98–2.00) ■	57.36	< 0.001
LC3-II/LC3-I	1.00 (0.66–1.33)	1.37 (0.96–1.90) ▲	0.89 (0.55–1.16) △	1.21 (1.12–1.59) ■	1.60 (1.26–1.93) †	1.70 (1.10–2.17)■	1.12 (0.98–1.33) △	1.58 (1.27–1.80) ♦	1.47 (1.05–1.58) ■ ♦	33.83	< 0.001
SQSTM1/p62	1.00 (0.55–1.57)	1.06 (0.88–1.31)	0.60 (0.48–0.77) ▲ △	0.60 (0.39–0.72)▲ △	0.66 (0.57–0.87) △	0.33 (0.19–0.40) ■ †	0.58 (0.42–0.78) ▲ △	0.63 (0.53–0.84) △	0.21 (0.19–0.38) ■ ♦	48.28	< 0.001
Rubicon	1.00 (0.50–1.73)	1.18 (0.76–2.26)	1.95 (1.37–2.42)	0.00 (0.00–0.17) ▲ △ ■	0.00 (0.00–0.24) △	0.00 (0.00–0.19) ■	0.00 (0.00–0.25) ▲ △ ■	0.00 (0.00–0.22) △	0.00 (0.00–0.18) ■	51.77	< 0.001
NOX2	1.00 (0.83–1.11)	0.93 (0.88–1.05)	1.04 (0.81–1.09)	0.42 (0.36–0.56) ▲ △ ■	0.35 (0.28–0.48) △	0.35 (0.30–0.62) ■	0.55 (0.51–0.63) ▲ △ ■	0.62 (0.55–0.65) △	0.62 (0.46–0.71) ■	37.78	< 0.001

Results are expressed as median and quartiles (in brackets) of protein/β-actin level normalized by vehicle median.

CQ, Chloroquine. Rapa, Rapamycin. AH, Aluminum oxyhydroxide. V, Whole vaccine (Engerix^®^).

▲*p* < 0.05 compared to vehicle treatment.

△*p* < 0.05 compared to CQ treatment.

■*p* < 0.05 compared to Rapa treatment.

^†^*p* < 0.05 compared to AH treatment.

♦*p* < 0.05 compared to V treatment.

These autophagy-competent PBMCs were exposed to AH or whole vaccine (V) during 4 h, with and without autophagy modulators. AH modulated autophagy proteins levels, increasing LC3-II and reducing SQSTM1/p62 compared to vehicle. AH + CQ treatment led to significantly greater amount of LC3-II and increased the LC3-II/LC3-I ratio compared to AH alone, indicating CQ inhibition of AH-induced autophagy. Compared to CQ treatment, AH + CQ increased the level of LC3-II and reduced the SQSTM1/p62 ratio assuming an autophagy activation (Table 1). Compared to AH treatment, LC3-II and SQSTM1/p62 were reduced after AH + Rapa confirming the induction action of Rapa. This treatment also increased LC3-II and LC3-II/LC3-I and reduced SQSTM1/p62 compared to the Rapa treatment pointing out a common autophagy activating effect of Rapa and AH on PBMCs (Table 1).

AH-containing V produced a reduction in SQSTM1/p62 compared to vehicle which could indicate an active autophagy flux (Table 1). Compared to V alone, V + CQ increased LC3-II level and the LC3-II/LC3-I ratio, indicating an inhibition of autophagy by CQ, as observed with AH + CQ. Compared to CQ treatment, SQSTM1/p62 was significantly reduced after V + CQ treatment assuming PBMCs response was similar following AH and V exposure (Table 1). Compared to V alone, V + Rapa increased the LC3-II/LC3-I ratio and reduced the SQSTM1/p62 ratio confirming Rapa autophagy induction. V + Rapa also increased LC3-II and the LC3-II/LC3-I ratio and reduced the SQSTM1/p62 ratio compared to Rapa alone suggesting that V as well as AH activates the autophagy pathway in PBMCs after only 4 h (Table 1).

**Figure 2:**
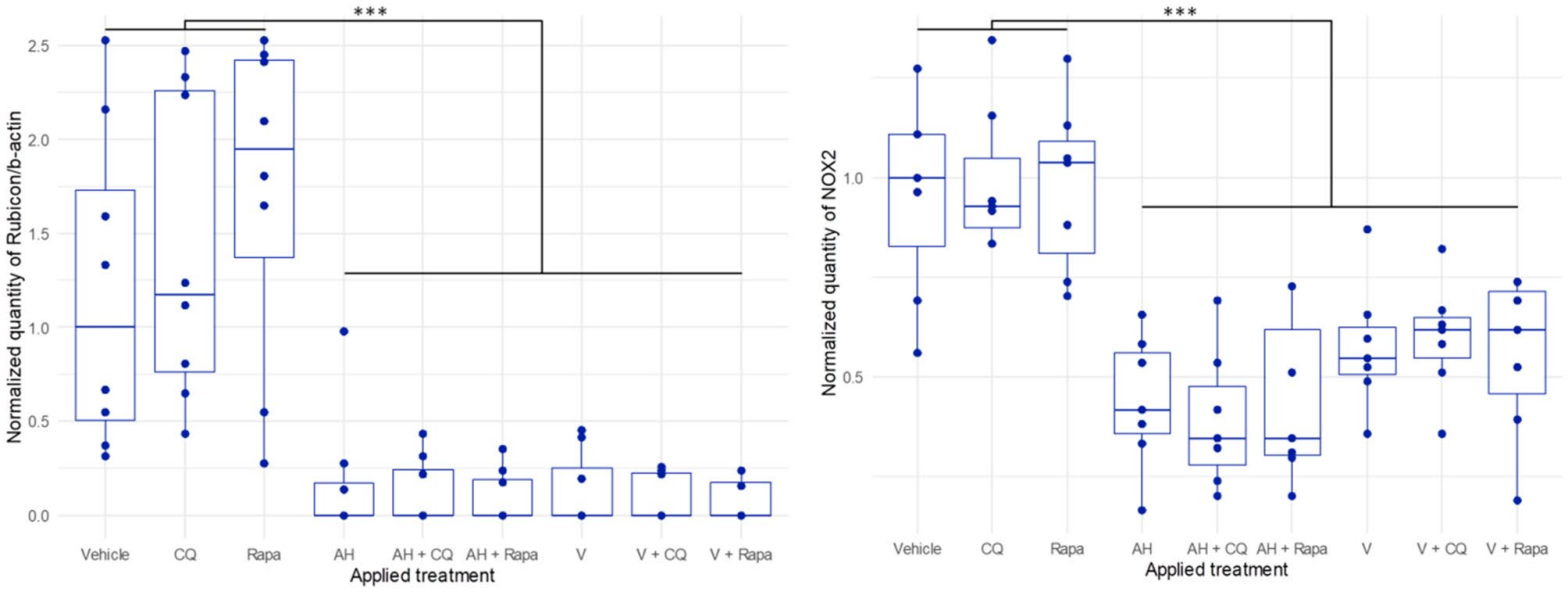
Expression level of Rubicon (left panel) and NOX2 (right panel) into differentiated PBMCs exposed 4 h with several treatments. CQ, Chloroquine, Rapa, Rapamycin, AH, Aluminum oxyhydroxide, V, Whole vaccine (Engerix^®^), *statistical differences at Durbin-Conover post-hoc test (***p < 0.001).

As expected, pharmacological modulators of classical autophagy did not affect expression of Rubicon and NOX2, two major components of the LAP pathway that are not autophagy-related proteins. Importantly, the presence of Al particles (AH or V) decreased the amount of Rubicon compared to vehicle. The same results were obtained when AH + CQ or V + CQ were compared to CQ alone and when AH + Rapa or V + Rapa were compared to Rapa alone (Table 1 and Fig. 2 left panel). Similarly to Rubicon, NOX2 level was greatly reduced in presence of Al particles (AH or V) compared to vehicle. This was also observed after AH + CQ or V + CQ treatment compared to CQ alone, and after AH + Rapa or V + Rapa treatment compared to Rapa alone (Table 1 and Fig. 2 right panel). Such reductions of Rubicon and NOX2 levels upon exposure to AH strongly suggest that LAP is implicated in the adjuvant internalization by immune cells, and, presumably, in its subsequent adjuvant action.

### AH-induced inflammatory response starts with TNF-α release

**Figure 3:**
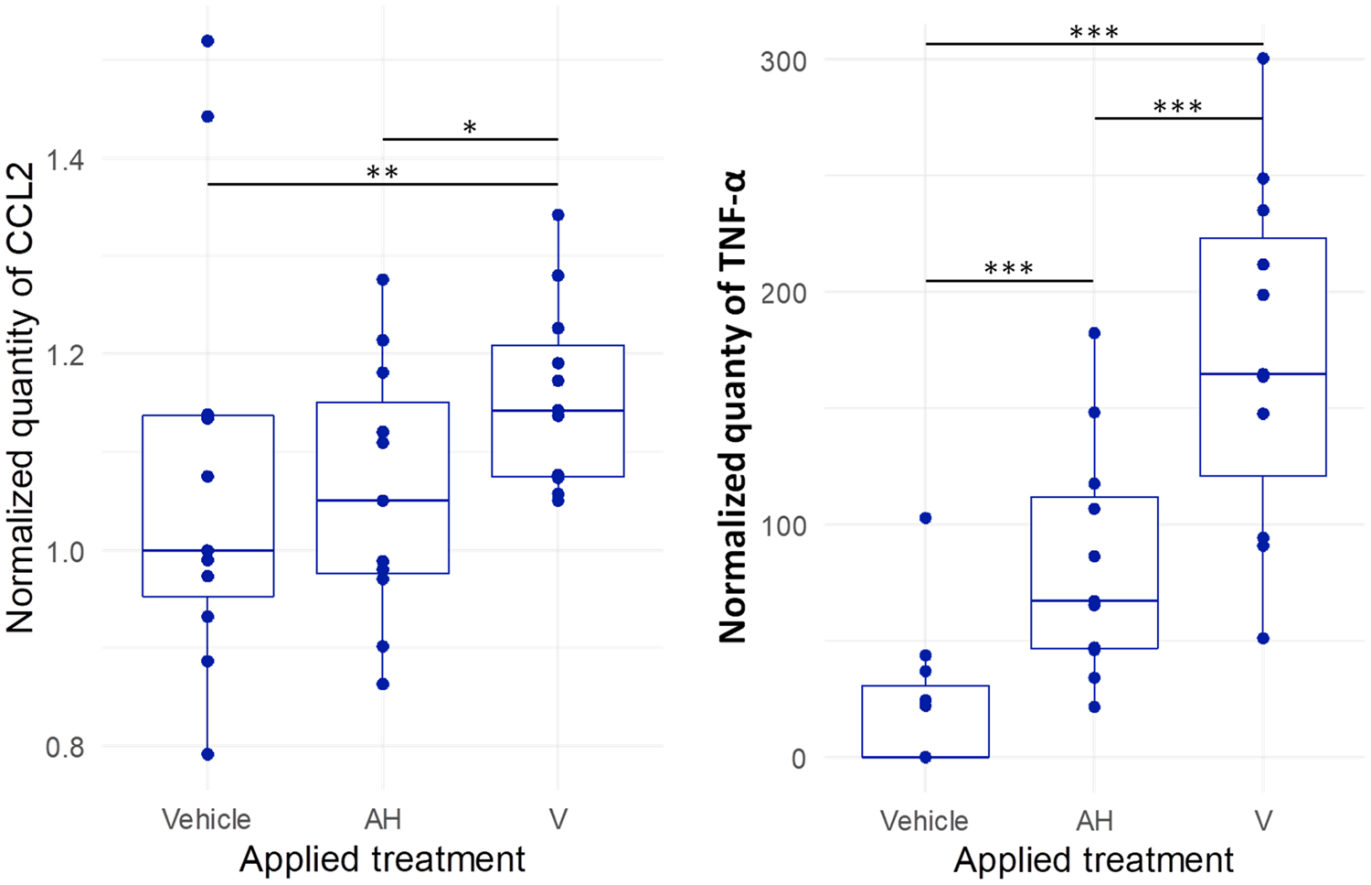
Expression level of CCL2 (left panel) and TFN-α (right panel) into differentiated PBMCs exposed 4 h Al-based treatments. AH, Aluminum oxyhydroxide. V, Whole vaccine (Engerix^®^). *statistical differences at Durbin-Conover post-hoc test (*p < 0.05, **p < 0.01, ***p < 0.001).

Cytokine quantification was done using the cell supernatant to explore the initial inflammatory signal induced by AH recognition and/or internalization by differentiated PBMCs. The inflammatory response was quite limited after 4 h of exposure. Four among the 11 studied cytokines (IL-1β, IL-12p40, IL-18, and TGF-β) were below the detection threshold. Among the 7 expressed cytokines, 5 were unaffected by AH or V exposure compared to vehicle control (IL-6, IL-8, IL-10, CXCL1, and CCL4) (Table 2). The 2 cytokines impacted by treatments were chemokine ligand 2 (CCL2) previously called monocyte chemoattractant protein 1 or MCP1, and TNF-α (Table 2). CCL2 was significantly overexpressed after 4 h of V treatment by comparison with vehicle or AH alone (Fig. 3, left panel). AH treatment did not affect CCL2 expression at 4 h compared to vehicle control, thus suggesting specific, or more pronounced, effect of antigen + AH compared to the adjuvant alone. TNF-α was the most importantly released cytokine after 4 h of treatment. A significant TNF-α increase was observed after both AH and V treatments compared to vehicle control. Comparing both treatment, V and AH, TNF-α secretion was significantly higher when cells were exposed to the V, containing AH and antigens, showing the effect of antigens in enhancing TNF-α secretion (Fig. 3, right panel). In summary, TNF-α appeared to be uniquely excreted by PBMCs during the early inflammatory response to AH, with or without antigens.

**Table 2:** Cytokine quantification into differentiated PBMCs exposed 4 h with Al-based treatments.

Treatment Cytokine	Vehicle	AH	V	Friedman	
				Χ^2^	sig
IL-1β	Only three samples above the detection threshold Statistics not applied				
IL-6	1.00 (0.48–2.29)	1.65 (0.77–1.94)	1.97 (1.08–2.61)	5.09	n.s
IL-8	1.00 (0.79–1.10)	0.93 (0.85–1.03)	1.09 (1.01–1.18)	5.64	n.s
IL-10	1.00 (0.23–1.32)	0.62 (0.00–2.222)	2.47 (0.53–3.32)	3.35	n.s
IL-12p40	No sample above the detection threshold Statistics not applied				
IL-18	Only four individuals above the detection threshold Statistics not applied				
CXCL1	1.00 (0.77–1.18)	0.99 (0.81–1.13)	0.97 (0.89–1.27)	4.55	n.s
CCL2	1.00 (0.95–1.14)	1.05 (0.98–1.15)	1.14 (1.08–1.21) ▲ △	7.09	0.029
CCL4	1.00 (0.71–1.17)	1.05 (0.75–1.16)	1.11 (0.82–1.30)	1.27	n.s
TGF-β	No sample above the detection threshold Statistics not applied				
TNF-α	0 (0–30.84)	67.07 (46.7–112.22) ▲	164.62 (121.05–223.65) ▲ △	20.18	< 0.001

Results are expressed as median and quartiles (in brackets) of cytokine pixel signal/internal positive control.

When possible, results were normalized by vehicle median.

AH, Aluminum oxyhydroxide. V, Whole vaccine (Engerix^®^). n.s*.,* not significant.

▲*p* < 0.05 to Durbin-Conover post-hoc test compared to vehicle treatment.

△*p* < 0.05 to Durbin-Conover post-hoc test compared to AH treatment.

### ROS production

The importance of ROS for macrophage-mediated immunity is unquestioned. Thus, we quantified production of both cytosolic and mitochondrial ROS in cells after 4 h of AH and V treatments. As a first step, the fluorescent probe (H_2_DCFDA), we used to visualize ROS, was tested in differentiated PBMCs exposed to H_2_O_2_ during 4 h. This positive control showed a significant enhancement of the signal assessing ROS production. The vaccine produced a small but statistically significant increase of ROS compared to vehicle. This increase was not observed in the case of AH during the first four hours (Table 3). Expectedly, both AH and V leaded to significantly more ROS production in the presence of H_2_O_2_ than without H_2_O_2_, but, quite surprisingly, they induced less important ROS production compared to H_2_O_2_ alone, suggesting possible reduction of H_2_O_2_ cytotoxicity by AH (Table 3).

**Table 3:** ROS (cytosolic and mitochondrial) quantification into differentiated PBMCs exposed 4 h with several treatments.

Treatment	Vehicle	AH	V	H_2_O_2_	AH + H_2_O_2_	V + H_2_O_2_	Friedman	
							Χ^2^	*p*
ROS	1.00 (0.71–2.35)	1.04 (0.54–1.95)	1.07 (0.47–1.91) ▲	1.99 (1.07–4.47) ▲ † ♦	1.36 (0.67–2.94) † △	1.24 (0.52–2.33) ♦ △	23.94	< 0.001

Results are expressed as median and quartiles (in brackets).

Units are standardization of the considered signal by protein quantity normalized by vehicle median.

AH, Aluminum oxyhydroxide. V, Whole vaccine (Engerix^®^).

▲*p* < 0.05 compared to vehicle treatment.

△*p* < 0.05 compared to H_2_O_2_ treatment.

†*p* < 0.05 compared to AH treatment.

♦*p* < 0.05 compared to V treatment.

### AH impairs maximal cellular oxygen consumption leading to saturation of the mitochondrial metabolism

Since mitochondrial metabolism generates ATP and must constantly adapt to stress conditions in order to maintain bioenergetic levels related to cellular functions, we evaluated, under different conditions, the PBMCs mitochondrial oxygen consumption rate (OCR) and spare respiratory capacity (SRC), two particularly robust functional tests assessing mitochondrial function and reserve.

**Figure 4:**
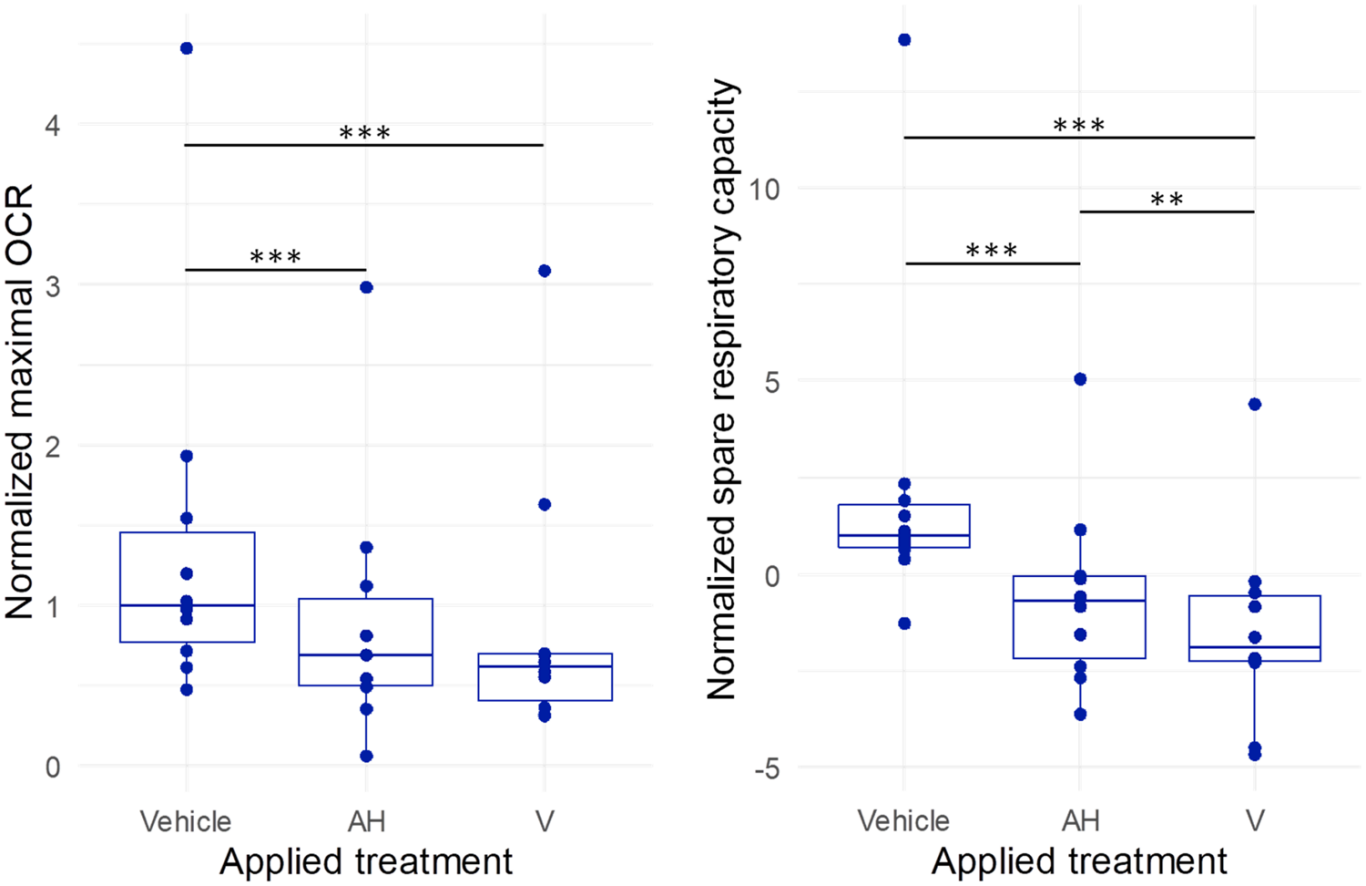
Measure of maximal OCR (left panel) and spare respiratory capacity (right panel) of PBMCs’s mitochondria exposed 4 h with Al-based treatments. AH, Aluminum oxyhydroxide. V, Whole vaccine (Engerix^®^). *statistical differences at Durbin-Conover post-hoc test (**p < 0.01, ***p < 0.001).

Differentiated PBMCs exposed to AH or V showed reduced maximal OCR after 4 h of contact (Fig. 4 left panel, Table 4). They also expressed a significant decrease of SRC (Fig. 4 right panel, Table 4). These metabolic alterations were associated with a small increase of proton leak that did not reach the significance threshold. Similar small increases were observed for basal OCR, and ATP production (Table 4). Taken together, these variations suggest that cellular exposure to AH, with or without antigen, induced a conspicuous energetic demand likely leading to spare respiratory capacity saturation limiting further mitochondrial metabolic adaptation.

**Table 4:** Mitochondrial parameters into differentiated PBMCs exposed 4 h with Al-based treatments.

Treatment	Vehicle	AH	V	Friedman	
				Χ^2^	*p*
Basal OCR	1.00 (0.76–1.88)	1.30 (0.87–1.67)	1.14 (1.02–1.65)	1.40	n.s
ATP related OCR	1.00 (0.80–1.79)	1.39 (1.01–1.99)	1.16 (0.98–2.20)	2.89	n.s
Max OCR	1.00 (0.77–1.46)	0.69 (0.51–1.04) ▲	0.62(0.41–0.70) ▲	15.00	< 0.001
Proton leak	1.00 (0.79–1.97)	1.27 (0.91–1.50)	1.47(1.14–1.85)	0.68	n.s
Spare respiratory capacity	1.00(0.68–1.81)	− 0.71(− 2.19to − 0.06)▲	− 1.90(− 2.25 to − 0.57) ▲ †	16.80	< 0.001

Results are expressed as median and quartiles (in brackets) of OCR–SRC/protein quantification level normalized by vehicle median.

AH, Aluminum oxyhydroxide. V, Whole vaccine (Engerix^®^). n.s., not significant.

▲*p* < 0.05 compared to vehicle treatment.

^†^*p* < 0.05 compared to AH treatment.

## Discussion

ABAs were initially added to vaccines to compensate low immunogenicity of highly purified antigens used instead of living and attenuated viruses to improve vaccine safety. Among the main alleged mechanisms of ABAs adjuvant effects, the so-called “depot-theory” was not confirmed by ad hoc experiments^6^, and two plausible theories remained, including direct stimulation of innate immunity by pro-inflammatory response^17–19^ and cytotoxicity induced by the crystalline structure of ABAs^27,28^.

In the present paper, using a fluorescent probe (lumogallion) we first confirmed that AH is quickly internalized by immune cells instead of remaining extracellular until its solubilization by chelating agents as wrongly believed for decades^36^. Cellular internalization of AH was previously documented both in vivo, in muscle macrophages infiltrates of vaccinated humans^37^ and in animal models of vaccine injection^38,39^, and in vitro*,* in human THP-1 cell line^40^, and, using electron microscopy in primary human PBMCs^41^. In the latter study, AH was internalized 48 h after treatment and remained intracellular at least a week after replacement of the medium. Using a 25-fold higher concentration of AH (*ie* 50 µg Al/ml), we observed that AH was already captured by PBMCs after 4 h of contact. These data indicate that ABAs are actively and rapidly engulfed by differentiated PBMCs and could exert a long-lasting action within immune cells afterwards. Moreover, the co-localization of lumogallion and lysotracker signals suggest that PBMCs use clearance mechanisms implicating lysosomes, namely macroautophagy (xenophagy) and/or LAP, thus handling AH particles agglomerates as pseudo-pathogens. Regarding the physico-chemical properties of AH, previous studies from our group showed that AH in both commercial suspensions or in vaccines represent a positively charged fiber-like particles^29,42^. However, it should be noted that a conversion of the charge, from negatively to positively charged, of AH particles in the culture medium has been reported by Mold et al*.*^43^. This conversion may be due to the presence of proteins in the culture medium. Moreover, the presence of the antigens in vaccines can modify the size of particles and lead to the formation of aggregates of larger particles, which can also have an effect on certain cellular mechanisms such as aforementioned LAP or autophagy and cellular cytotoxicity^29^. In addition to the presence of antigens, the dilution has a real impact in the size of particles and the greater the dilution is, the more the aggregates will dissociate to form smaller particle sizes^29^. So, the size of particles in the whole vaccine (500 µg Al^3+^/ml) differs than the size of particles in the diluted solutions (50 µg Al^3+^/ml).

As assessed by western blotting, expressions of autophagy and LAP proteins were modulated by AH, with or without antigens. To briefly describe the context, autophagy and LAP are both clearance mechanisms sharing common autophagy related proteins such as PI3KCIII complex or ATG12-ATG5–ATG16 complex^44^. The main difference between autophagy and LAP consist in where the initial vesicle is formed. Autophagosome is an intracytoplasmic vesicle able to sequester defective proteins and organelles when LAPosome is a vesicle formed on the cell membrane to internalize extracellular compounds^44^. At the protein level, LAP is distinguished from autophagy by Rubicon which is a protein able to bind the PI3KCIII complex on the LAPosome membrane and switch its activator capability from autophagy to LAP^44,45^. This activation of LAP leads to NOX2 recruitment and subsequent LAPosome degradation. For more details about convergence and divergence between autophagy and LAP, see Heckmann and Green review^45^.

Autophagy activation was assessed by combined LC3 up-regulation and SQSTM1/p62 downregulation as described by Klionsky et al*.*^35^. LAP proteins down-regulation suggested involvement of this specific phagosomal pathway in recognition and internalization of AH. Autophagy activation has been observed in dendritic cells (DCs) following exposure to Alpha-alumina nanoparticles (Al_2_O_3_-Ovalbumin)^46^ and in macrophages following AH exposure^41^. The observed activation of both autophagy and LAP flux suggests that the bulk of adjuvant could have been internalized by LAP and that a part escaped the phagosome due to phagosomal membrane destabilization, leading to the inflammasome activation^30,47^. However, the elementary particles of AH (i.e. nanoparticles < 20 nm) are able to diffuse through the membrane without the contribution of an endocytotic mechanism^48^. Two mechanisms can therefore lead to the entry of membrane unbound AH particles in the cytoplasm, the presence of which has been previously assessed by electron microscopy in vivo^37^, and activate autophagy as an attempt to clear out the undesirable and potentially noxious alien. Importantly, the activation of autophagy and LAP could be unbalanced since up-regulation of the LAP-specific Rubicon is known to modulate the autophagy/LAP common cellular machinery from autophagy to LAP^44^. Negative regulation of autophagy by LAP activation could lead to reduced effectiveness of intracellular adjuvant clearance explaining the phagosomal pathway obstruction observed in THP-1 cells after AH exposure^49^, likely participating to long-lasting adjuvant bio-persistency and the related inflammatory response.

Indeed, ABAs are known to produce a strong inflammatory response characterized by NRLP3 inflammasome activation and subsequent pro-inflammatory cytokine release. Moreover, dysregulation of autophagy that is a regulator of NLRP3 inflammasome proteins able to contain the inflammatory response^50^, could participate to this strong inflammatory answer. It has been claimed that IL-1β and IL-18 production by human PBMCs or DCs in response to ABAs should reflect caspase-1 production^17^, the main effector of the NRLP3 inflammasome^18,19^. In our short duration study, however, IL-1β and IL-18 were not detected; only two cytokines (CCL2 and TNF-α) being excreted by PBMCs in response to AH or vaccine.

Up-regulation of CCL2 is responsible for circulating monocytes influx participating to the local immune response. In addition, AH-containing particle in vivo tracking and gain/loss of function experiments have clearly documented the central role of CCL2 as a driver of significant particle-loaded immune cells spreading from the injection site to the draining lymph nodes and spleen^51^. In the present study, CCL2 excretion was enhanced by vaccine but not by AH alone 4 h after exposure. Consistently, an in vivo study has previously reported no change of CCL2 gene expression 72 h after intramuscular injection of AH in mice^52^. Our results indicate that monocytes recruitment at the vaccination site may not be induced by the adjuvant per se but rather by vaccine antigens or by the combination of antigens with AH.

In contrast to CCL2, TNF-α excretion was significantly enhanced by both vaccine and AH alone, the strongest TNF-α response being induced by vaccine. In vivo, TNF-α up-regulation following AH administration has been shown to be transitory, being no longer detected in mouse muscle 16 h after injection^52^.

Although kinetic studies and extensive profiling are needed to fully characterize early cytokine production by human PBMCs upon AH or vaccine exposure, TNF-α appears to be a choice candidate as the first up-regulated inflammatory mediator. The lack of IL-1β and IL-18 detection within 4 h of AH exposure despite the well-established AH activation of the NLRP3 inflammasome assessed that the quick and transitory AH-induced TNF-α production preceded inflammasome activation. Interestingly TNF-α has been shown to be a strong inducer of inflammasome at the transcriptional level^53^ and to promote particulate matter-mediated inflammation via the NLRP3 inflammasome^54^. Further studies are warranted to substantiate such an instrumental role of TNF-α in the specific context of ABAs adjuvant effects.

We also examined if a ROS-dependent mechanism acting in parallel with TNF-α may trigger inflammasome activation^55^. Indeed, ROS are an important component of the immune response to pathogen phagocytosis via activation of NOX2 which generates superoxide anions in the phagosome^56^, and, in addition, ROS may be potentially generated by AH-induced mitochondrial impairment, directly or indirectly. In the present study, ROS production was not significantly increased after 4 h of AH exposure and was only slightly increased with vaccine, suggesting again an enhanced biological effect of antigens adsorbed at the surface of AH compared to AH alone. Since ROS production is usually slow but sustained^57^ our analysis could have been inappropriately short to detect a signal using a fluorescent probe. Indeed, this technique seems to detect ROS overproduction only after 24 h in DCs treated with zymozan (a strong LAP inductor leading to NOX2 activation and ROS generation)^57^. Thus, no conclusion could be reached until longer experiments are made. We would like to call for caution about possible cross-reactions between AH and H_2_O_2_ used as positive control. Indeed, AH could adsorb H_2_O_2_ limiting its oxidative capacity in living cells^58^ and our results consistently suggested that AH could limit H_2_O_2_ induced ROS production.

Our exploratory evaluation of the mitochondrial metabolism showed a tendency of AH to increase basal mitochondrial metabolism, seemingly linked to energetic request, ATP production being necessary to support phagocytosis and cytokine production, as recently described^59^. It also showed that AH and vaccine impaired both the maximal respiratory capacity of mitochondria, probably in accordance with increasing proton leak, and spare respiratory capacity assessing mitochondrial reserve. It could be contributory to examine if and how the mitochondria of primary PBMCs are morphologically impacted by AH, since a 8–72 h exposure of THP1 cells to AH apparently could induces mitochondria fission alteration more than membrane integrity damage^49^. Both increased energetic demands and maximal respiratory capability impairment could have resulted in the observed default of mitochondria spare capacity in the presence of AH and vaccine. These results may predict that the previously documented long-term bio-persistence of AH within immune cells^37^ could impact mitochondrial function in a way reducing their ability to adapt energy production to future cell needs.

In conclusion, this paper confirms current knowledge on ABAs implicating inflammatory response and oxidative stress, using human primary immune cells. However, considering the possible interconnexion between the different cell components studied, early cellular events occurring upon exposure to aluminum oxyhydroxide adjuvant could imply cross-reactions between several factors such as LAP/Autophagy, inflammation, oxidative stress, and mitochondrial metabolism. Indeed, data showed that AH is quickly recognized and internalized by immune cells and activates both LAP and autophagy in the first 4 h of contact. This double clearance mechanism activation indicates that ABAs are handled by immune cells like extracellular (LAP) and intra-cytoplasmic (autophagy) pseudo-pathogens. As previously described AH promotes an inflammatory response, the response being enhanced by adsorbed antigens, and starts with TNF-α. Adjuvant recognition, internalization, and clearance attempts require energy provided by ATP generated by mitochondria. However, mitochondrial metabolism is affected by AH which limits their maximal respiratory capabilities and leads to decreased mitochondrial reserve thus limiting the possibility of cells to adequately respond to subsequent energetic challenges. Considering that autophagy regulates inflammasome components^50^ and recycle defective mitochondria^60^, any dysfunction of this core homeostatic mechanism could become deleterious following an exposition to AH.

Our exploratory paper clearly points out that future research on ABAs adjuvant effects must investigate the kinetics of clearance mechanisms, inflammatory response and mitochondrial functions in an integrated multi-systemic approach, with special emphasis put on new avenues including: (1) LAP implication in the adjuvant handling; (2) mechanisms triggering early TNF-α production; and (3) short and long-term impact on mitochondrial function, integrity, biogenesis and turn-over.

## Methods

### Human participants

Written informed consent was obtained from all persons included in the study, after the nature and possible consequences of the studies had been fully explained, in accordance with the *Declaration of Helsinki* protocols, and our experimental protocols were approved by the ethic “*Person Protection Committee*” (IRB approval 2012, CPP Ile-de-France Paris 11). All blood samples were obtained from the French Blood Establishment (Etablissement Français du Sang—EFS) according to the covenant with the Paris Est University Hospital Henri Mondor, Créteil, France (# C CPSL UNT—N°18/EFS/033).

Blood sample were collected in a total of 14 healthy female participants (median and quartile age: 48 (43–52.25)). According to the available amount of cells, 11 (age: 48 (48–53.5) years) were used for internalization, inflammatory response, and autophagy evaluation and 10 (age: 49 (39.75–53.75) years) for ROS production and mitochondrial metabolism with an overlap of 8 individuals between the two experimental phases.

### Blood collection

After delivery, whole blood was immediately layered on Ficoll pillow and centrifuged at 500 Relative Centrifugal Force (RCF) without break for 20 min. The Peripheral Blood Mononuclear Cells (PBMCs) layer was then separated and washed with phosphate buffered saline (PBS) and diluted at a final concentration of 20.10^6^ cells per ml in freezing solution made from 95% of Fetal Bovine Serum (FBS) and 5% of DiMethylSulfOxyde (DMSO). PBMC suspensions were frozen at − 80 °C over-night then stored in liquid nitrogen until analyzed.

### Cell culture

The PBMC suspensions were quickly thawed on 37 °C *Dulbecco’s Modified Eagle Medium* (DMEM) then centrifuged at 300 RCF for 5 min and wash in PBS. After a second centrifugation, PBMCs were suspended in RPMI 1640 + Glutamax with 1% of penicillin/streptomycin and 1% L-glutamin 200 mM. Cells were cultured 3 h for adherence at a final density of 400.10^3^ cells per cm^2^ in 37 °C and 5% CO_2_ on different plates depending of the analysis. Phagocytosis observation was performed in Ibidi blade, cytokines assay and western blot in 24 wells culture plates, ROS assay in 96 wells culture plates, and mitochondrial metabolism evaluation in Seahorse XFe24 culture plates. After 3 h of adherence, culture medium was gently removed and replaced by a 37 °C differentiation medium composed of RPMI 1640 ATCC Modification with 1% of penicillin/streptomycin, 10% of FBS, and 0.1% of human *Macrophage Colony Stimulating Factor* (M-CSF) and *Granulocyte–Macrophage Colony-Stimulating Factor* (GM-CSF). After 6 days of differentiation, half of the culture medium was replaced for another 24 h by freshly prepared medium. Analyses were performed after a full week of differentiation and exposure to several kind of Al-based particles, such as AH and whole Al-based vaccine previously characterized by our research team in term of size, charge and shape^29,42^.

### Al engulfment observations

Internalization of Al particles was observed using lumogallion-stained Al particles. This fluorescent Al was produced following a previously published method^61^. Briefly, an AH commercial solution (Invivogen, vac-alu-250), diluted at 5 mg Al/ml in RPMI 1640 ATCC Modification medium was mixed overnight under sterile conditions and at room temperature with 50 µM of lumogallion on a rocking table. The day after, the pre-stained Al particles were collected by centrifugation for 10 min at 20 000 RCF. Finally, they were re-suspended in 1 ml RPMI 1640 ATCC Modification medium for a final concentration of 5 mg Al/ml and stored at +4 °C until use.

After a week of differentiation on Ibidi blade, PBMCs were treated 4 h with lumogallion-stained-Al at a final concentration of 50 µg Al/ml. In addition of fluorescent Al, cells were also tagged 45 min with Hoechst 33,342 and LysoTracker green DND-26 according to the manufacturer recommendations to stained nucleus and lysosomes.

Ibidi blades were observed on Zeiss Axio Observer Z1 microscope with 63X objective allowing phase contrast microscopy and fluorescent microscopy. For each Ibidi well, a minimum of 10 pictures were taken in order to obtain at least 50 analyzed cells per individual. Microscopy images were analyzed by Icy Software (V2.1.4.0 BioImage Analysis unit, Institut Pasteur, France)^62^. For each picture, cells were defined and for each cell the total fluorescence intensity of lumogallion and LysoTracker was calculating by addition of pixel intensity and normalized by cell surface to obtain mean intensity fluorescence per µm^2^. The degree of colocalization between fluorophores was calculated with Colocalization Studio package of Icy Software by pixel based evaluation of the Pearson correlation coefficient.

### Western blots

After the week of pre-treatment on 24 wells culture plate, differentiated PBMCs were exposed 4 h to vehicle (PBS), or to AH diluted to a final concentration of 50 µg Al/ml or to a whole vaccine (Engerix^®^ 20, GSK) adjuvanted on AH (500 µg AL/ml) with the appropriate dilution to obtain the same final concentration of 50 µg Al/ml. Positive and negative controls were obtained by using autophagy modulators that were respectively Rapamycin (Rapa) at 100 nM or Chloroquine (CQ) at 100 µM. Autophagy modulators were used 1 h prior and all along the Al treatments.

After the 4 h of treatment, cells were washed two times with cold PBS then lysed in 100 µl of 95 °C-pre-heated TR3 solution. The TR3 lysis solution is an aqueous solution of SDS at 170 mM, disodium phosphate at 10 mM, sodium orthovanadate at 1 mM, β-glycerophosphate at 10 mM, sodium pyrophosphate tetrabasic at 2.5 mM, sodium fluoride at 50 mM, 10% of glycerol, and cOmplete™ Mini Protease Inhibitor cocktail according to the manufacturer recommendations (Roche). Proteins extracts were sonicated 10 s then the total amount of protein was measured using a BCA Pierce™ protein assay kit.

For each individual, a normalized 14 µg of proteins was electrophoresed on 4–12% Bis–Tris Mini Protein Gels. Proteins were then electrotransferred to polyvinylidene difluoride membranes. Membranes were blocked in tris buffered saline (150 mM NaCl, pH 8.0) containing 0.1% Tween 20 (TBST) and 2.5% cold water fish skin gelatin for 60 min at room temperature with gentle agitation.

After blocking, membranes were then probed at +4 °C overnight with primary antibodies [rabbit anti-LC3B (1:1000; Life Technology, PA1-46286), rabbit anti-SQSTM1/p62 (1:3000; Life Technology, PA5-20839), rabbit anti Rubicon (1:1000; Ozyme, 8465S)]. Following primary antibody incubation, the membranes were washed 30 min in TBST and incubated with a beta-actin HRP conjugate (1:10,000; Santa Cruz, sc-47778HRP), a NOX2 HRP conjugate (1:500; Clinisciences, ORB223721) or an HRP-conjugated anti-rabbit secondary antibody at room temperature for 1 h then washed again for 30 min.

Results were visualized and recorded by chemiluminescence. Protein bands were quantified by ImageJ© software (V1.53i, Wayne Rasband and contributors, National Institute of Health, USA)^63^ and normalized to β-actin, which served as an internal control.

Autophagic function was assessed via changes in the expression of commonly used proteins (LC3, p62) and LAP function via specific components (Rubicon, NOX2). LC3 and especially LC3-II and SQSTM1/p62 are generally associated to autophagosomal membrane and used as marker of this cellular compartment presence. LC3-II/LC3-I ratio is usually a good indicator of autophagy activation state^35^.

### Cytokines assay

The same cell cultures performed for western blot were used for cytokines assay. After 4 h of treatment (described previously in the western blot section), the culture medium was used to perform a semi-quantification of an 11 excreted cytokines panel: interleukin (IL)-1β, IL-6, IL-8, IL-10, IL-12p40, IL-18, CXCL1, CCL2, CCL4, TGFβ, and TNF-α. Medium was stabilized by cOmplete™ Mini Protease Inhibitor cocktail to avoid protein degradation then incubated at +4 °C overnight on custom membranes from CliniSciences RayBio C-Series according to the manufacturer recommendations. Following the proteins capture, membranes were washed, incubated with biotinylated antibody, washed again then tagged with HRP-streptavidin available in manufacturer kit.

Results were pictured by chemiluminescence using the manufacturer substrate. Cytokines dots were quantified by Icy Software (V2.1.4.0 BioImage Analysis unit, Institut Pasteur, France)^62^ and normalized to positive controls included to membranes.

### ROS assay

After the 7 days of differentiation on 96 wells culture plate, PBMCs were exposed 4 h to vehicle, to AH or to the vaccine diluted at the same final concentration of 50 µg Al/ml. Positive controls were obtained by H_2_O_2_ treatment 45 min at 500 µM before data collection. ROS were stained by addition of H_2_DCFDA 45 min at 5 µM simultaneously with positive controls.

Results were visualized using a fluorescent plate reader allowing fluorescence readings at 530 nm after excitation at 485 nm along a matrix of 5 × 5 on each wells surface. After data recording, cells were lysed in 100 µl of 95 °C TR3 solution then the total amount of protein was measured using a BCA protein assay kit. Fluorescence of H_2_DCFDA was finally normalized by the total amount of proteins of each well.

### Mitochondrial metabolism evaluation

Differentiated PBMCs were exposed 4 h to AH or to the vaccine diluted at the same final concentration of 50 µg Al/ml. After Al treatments, cells were washed and kept in seahorse buffer along the measurement. Briefly, Seahorse buffer is an aqueous solution of EGTA (1 mM), MgCl_2_ (5 mM), KH_2_PO_4_ (10 mM), Mannitol (220 mM), Sucrose (70 mM), Glutamate (10 mM), Malate (2 mM), HEPES (2 mM), Pyruvate (10 mM), BSA (0.2%), and ADP (2 mM).

Oxygen consumption rate (OCR) was determined over 5-min increments. Three measures of stabilized OCR were taken before the sequential addition of the following compounds, each followed by three measures of OCR: 10 mM of succinate (Complex II substrate); 2.5 µM of oligomycin (ATP synthase inhibitor), 1 µM FCCP (an uncoupling protonophore), and 2.5 µM of antimycin A (Complex III inhibitor). Immediately after OCR record, cells were lysed in 100 µl of 95 °C TR3 solution then the total amount of protein was measured using a BCA protein assay kit. OCR measurements were standardized by the total protein amount of each well.

Several respiratory parameters were calculated, including basal respiration (succinate OCR–antimycin A OCR), ATP-linked respiration (succinate OCR–oligomycin OCR), proton leak respiration (oligomycin OCR–antimycin A OCR), maximal uncoupled respiration (FCCP OCR–antimycin A OCR), and spapre-capacity (FCCP OCR–succinate OCR).

### Statistical analysis

Data obtained from the presented experiments were all analyzed by Jamovi V2.3.12^64^ and graphical presentations were built with ggplot2 R package.

According to the relatively low number of data, non-parametric tests were used.

Friedman test followed by a Durbin-Conover pairwise post-hoc test when necessary were performed to compare treatments effect on western-blot, cytokine assays, ROS production, and mitochondrial variables.

All reported significance levels represent two-tailed *p*-values and critical alpha was set at 0.05 to indicate statistical significance.

## Supplementary Information

Supplementary Information.

## Data Availability

The datasets used and/or analyzed during the current study are available from the corresponding author after reasonable request.

## Abbreviations

ABA: Aluminum-based adjuvant
AH: Aluminum oxyhydroxide
Al: Aluminum
APC: Antigen-presenting cell
CCL2: Chemokine ligand 2 previously called monocyte chemoattractant protein 1 or MCP1
CQ: Chloroquine
DC: Dendritic cell
GM-CSF: Granulocyte–Macrophage colony-stimulating factor
IL: Interleukin
LAP: LC3-Associated phagocytosis
M-CSF: Macrophage colony stimulating factor
OCR: Oxygen consumption rate
PBMC: Peripheral blood mononuclear cell
Rapa: Rapamycin
RCF: Relative centrifugal force
ROS: Reactive oxygen species
SRC: Spare respiratory capacity
TBST: Tris buffered saline with 0.1% of tween 20
TNF-α: Tumor necrosis factor alpha
V: Whole vaccine (Engerix^®^)
