# Supplementary material for: Advances on the early cellular events occurring upon exposure of human macrophages to aluminum oxyhydroxide adjuvant
Source: Sci Rep. 2023 Feb 23;13:3198. doi: 10.1038/s41598-023-30336-1 (PMC9950428; doi:10.1038/s41598-023-30336-1)
Supplement: Supplementary file 1 — Supplementary Information. [file 41598_2023_30336_MOESM1_ESM.docx]

A


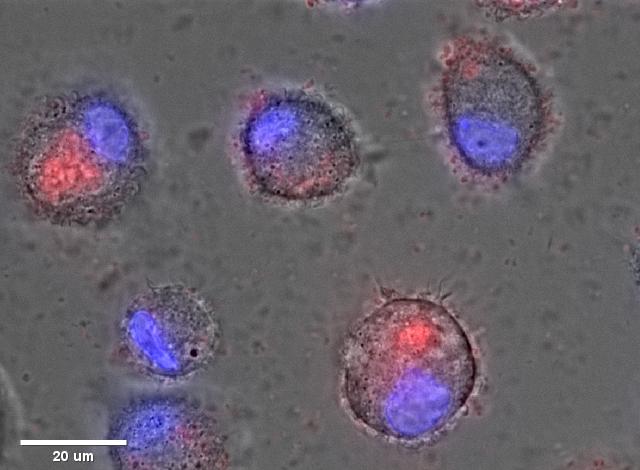

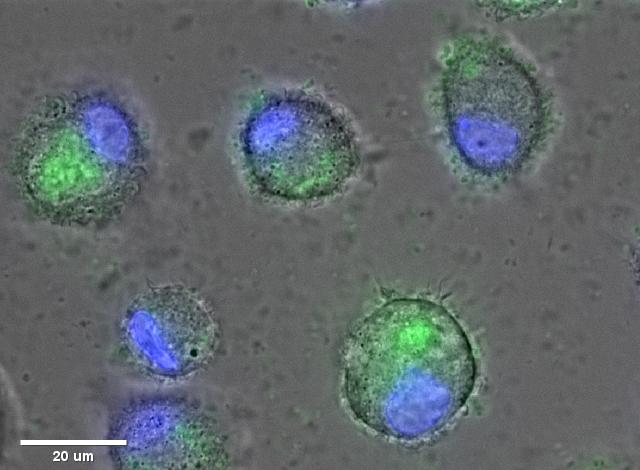

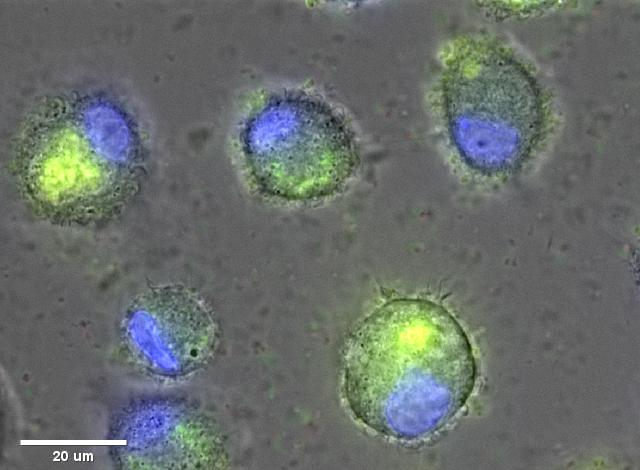


B


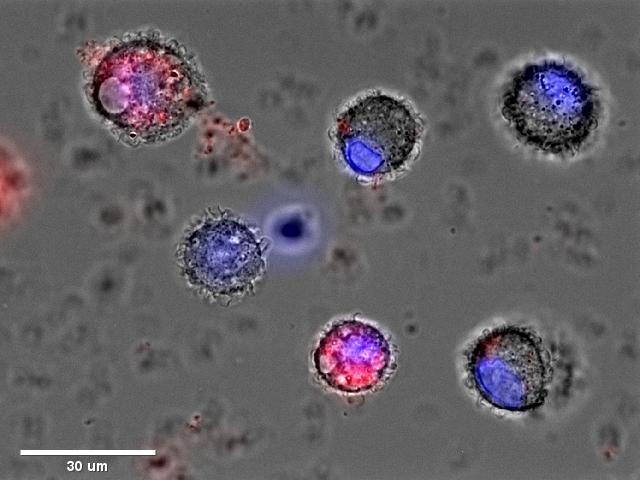

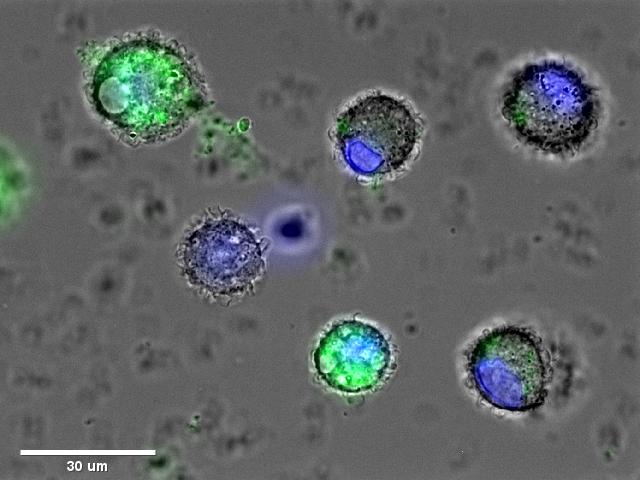

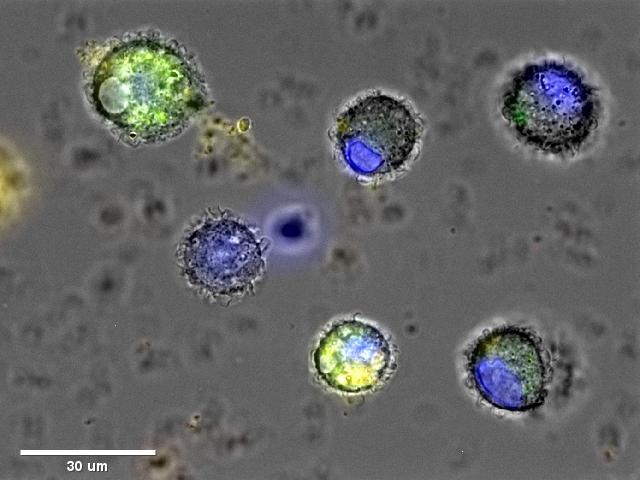


C


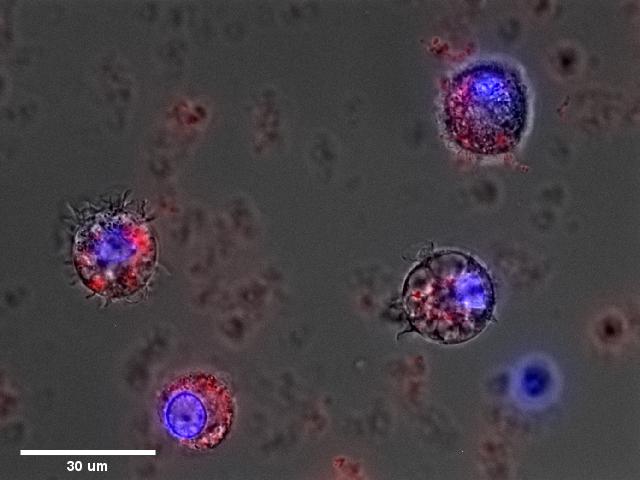

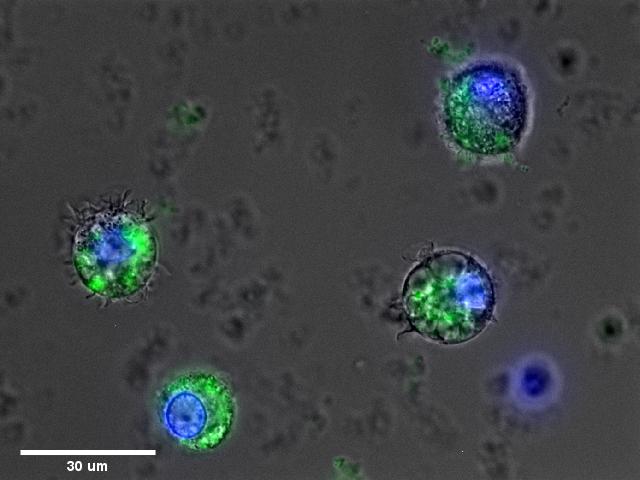

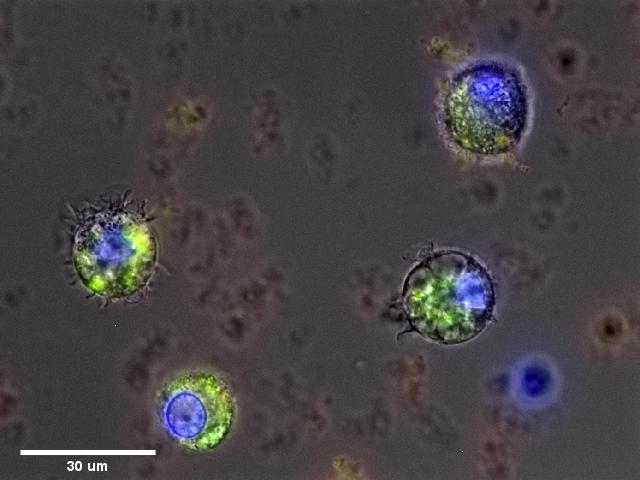


D


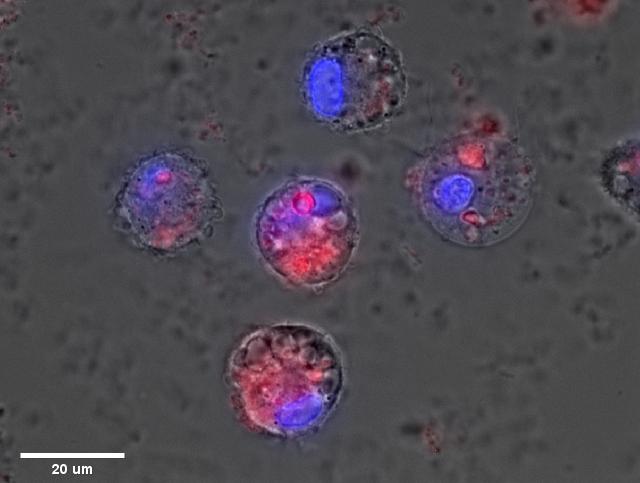

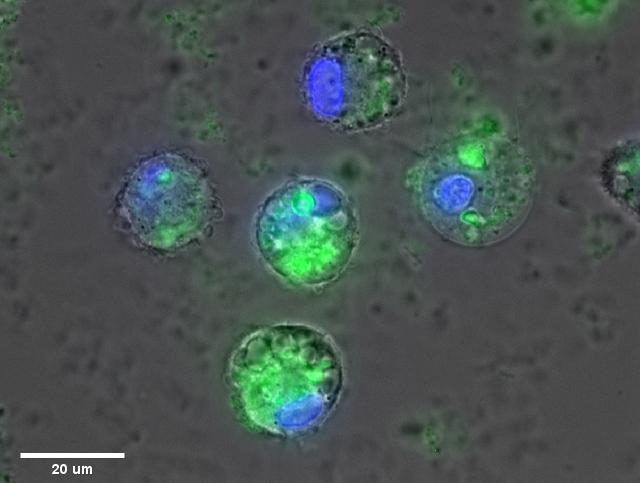

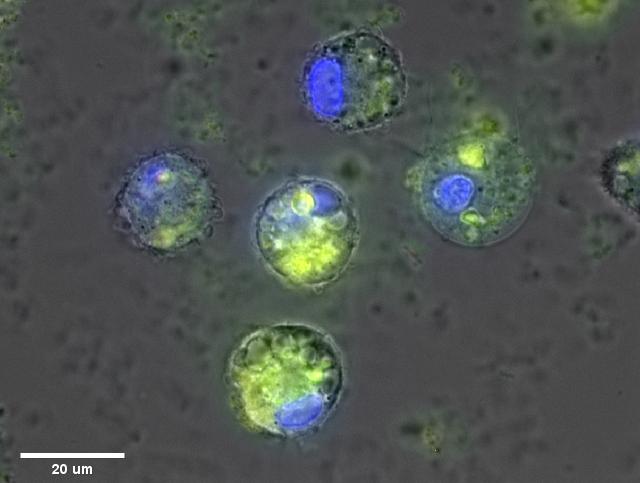


E


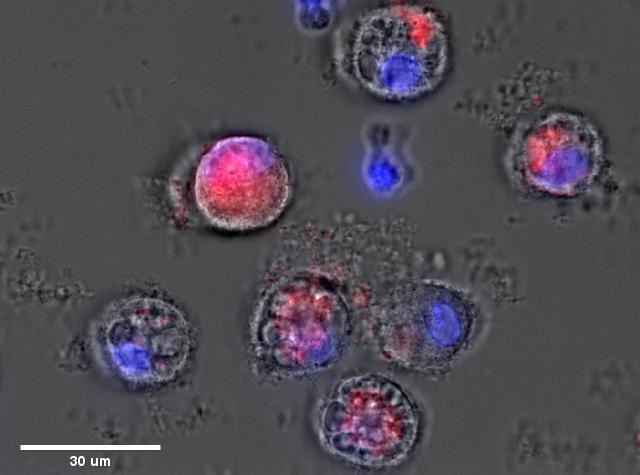

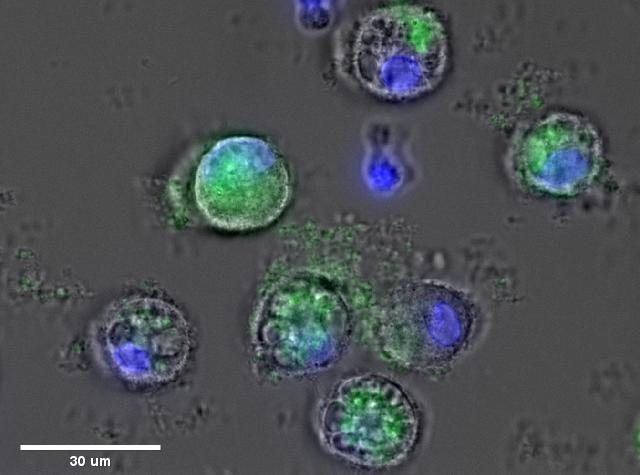

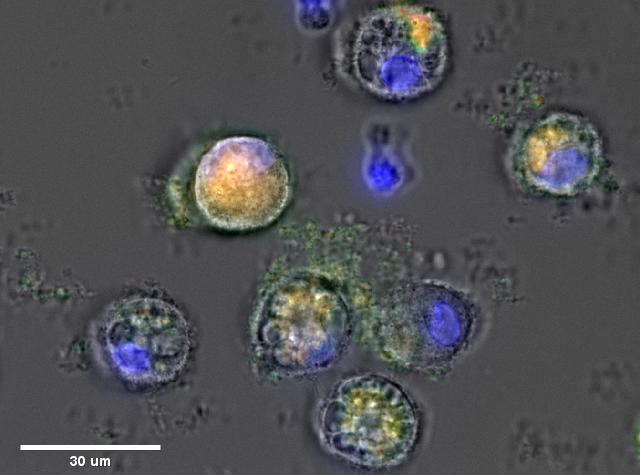


Supplementary 1: Representative panel of fluorescence microscopies showing lumogallion signal in red (left panel), lysotracker signal in green (central panel), and merged signals (right panel) into differentiated PBMCs of 5 individuals exposed 4 hours with lumogallion-stained-AH. Scale bars: (A, D; 20 µm), (B, C, E; 30µm).
